# Sphenoid Lymphoma: A Diagnostic Challenge

**DOI:** 10.7759/cureus.41058

**Published:** 2023-06-27

**Authors:** Shasikala Suthersan, Chenthilnathan Periasamy, Khairul Bariah Noh, Suk Kam Lee, Salina Husain

**Affiliations:** 1 Department of Otorhinolaryngology - Head and Neck Surgery, Universiti Kebangsaan Malaysia Medical Centre, Kuala Lumpur, MYS; 2 Department of Otorhinolaryngology, Penang General Hospital, Penang, MYS; 3 Department of Otorhinolaryngology - Head and Neck Surgery, Hospital Sultanah Bahiyah, Alor Setar, MYS; 4 Department of Pathology, Penang General Hospital, Penang, MYS

**Keywords:** sphenoid sinus lesions, endoscopic sinus surgery, lymphoma, sphenoid sinus, paranasal sinus

## Abstract

Sphenoid sinus lesions grasp the attention of Otorhinolaryngologists due to their prime location and vital surrounding structures. Once detected, these lesions require prompt investigation to identify the underlying cause, usually attributed to a tumor, fungal infection, sinusitis, or polyps, thus allowing tailored treatment.

We report a case of an elderly lady whose neurological presentation lead to the diagnosis of sphenoid sinus lymphoma. We discuss the diagnostic challenge in view of its interesting presenting symptoms as well as the surgical approach risk and limitations.

## Introduction

Primary extranodal lymphoma in the paranasal sinus is generally uncommon and accounts for only 8% of all sinonasal malignancies [1]. Lymphomas are malignancies of the lymphoreticular system, with large B cell lymphoma being the commonest type of non-Hodgkin lymphoma [2]. It is usually rare for lymphoma to arise primarily from a bony structure like the sphenoid sinus without visceral or lymph node involvement. It constitutes 3.1% of malignant bone tumors and 5% of extranodal lymphomas [2]. We note the aggressive behavior of lymphoma when it involves the paranasal sinus and the extensive invasion of vital surrounding structures. This further emphasizes the importance of early detection and diagnosis of sphenoid lesions, especially malignancies like non-Hodgkin lymphoma.

## Case presentation

**Figure 1:**
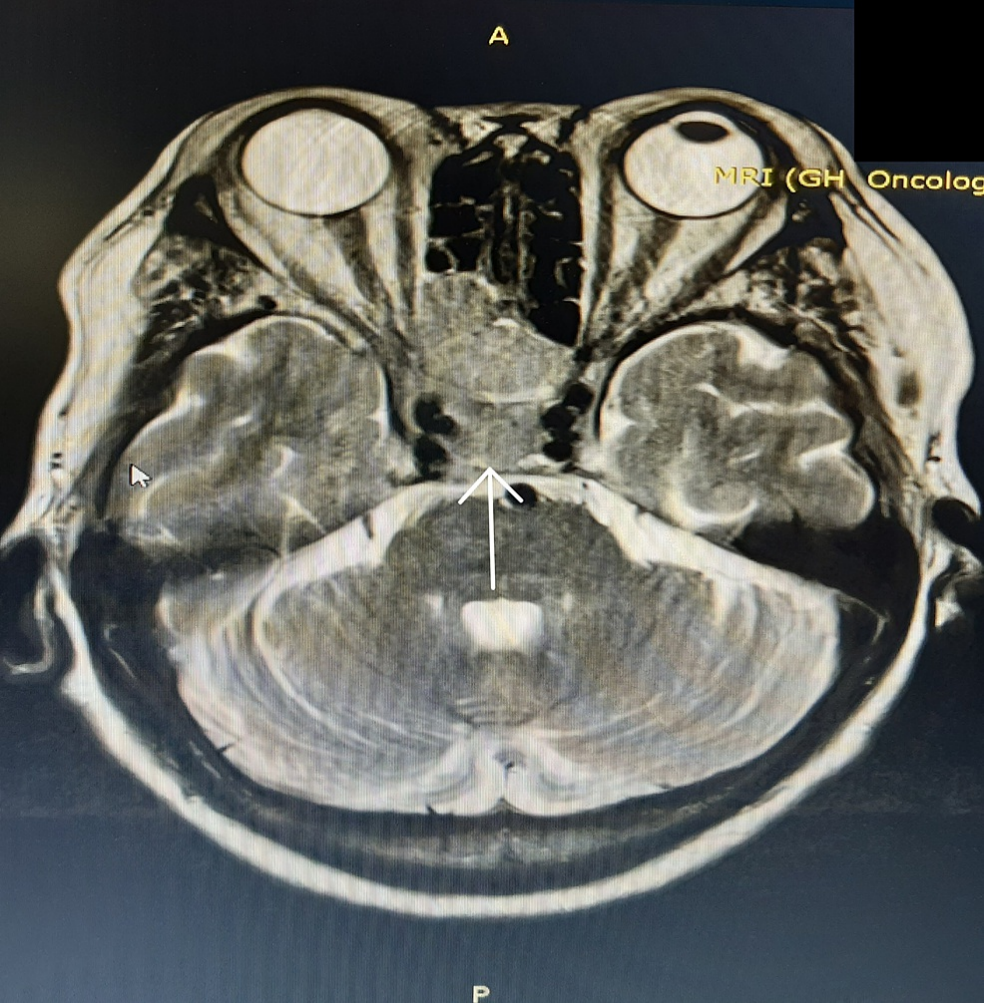
Tumor mass (white arrow) is more prominent on the right, extending to the ethmoidal sinus.

A 66-year-old female presented with a progressive right-sided frontal and temporal headache, blurred vision in her right eye, and numbness in the right side of the face, all of which had been developing over a period of three weeks. These symptoms were not accompanied by vomiting, dizziness, or any nasal symptoms such as anosmia, nasal blockage, nasal discharge, or epistaxis. On assessment, pain and touch sensation were absent on the right cheek. Nasal endoscopy showed normal findings. There was an absent sensation at the V2 distribution of the trigeminal nerve. CT scan of the paranasal sinuses showed soft tissue mass in the sphenoid sinus with intracranial extension. MRI of the brain and orbits showed a large enhancing tumor mass measuring about 5.0 x 3.2 x 2.5 cm in the sphenoidal sinus, more prominent on the right, with anterior extension into the ethmoidal sinuses and clival bone (Figure 1). 

**Figure 2:**
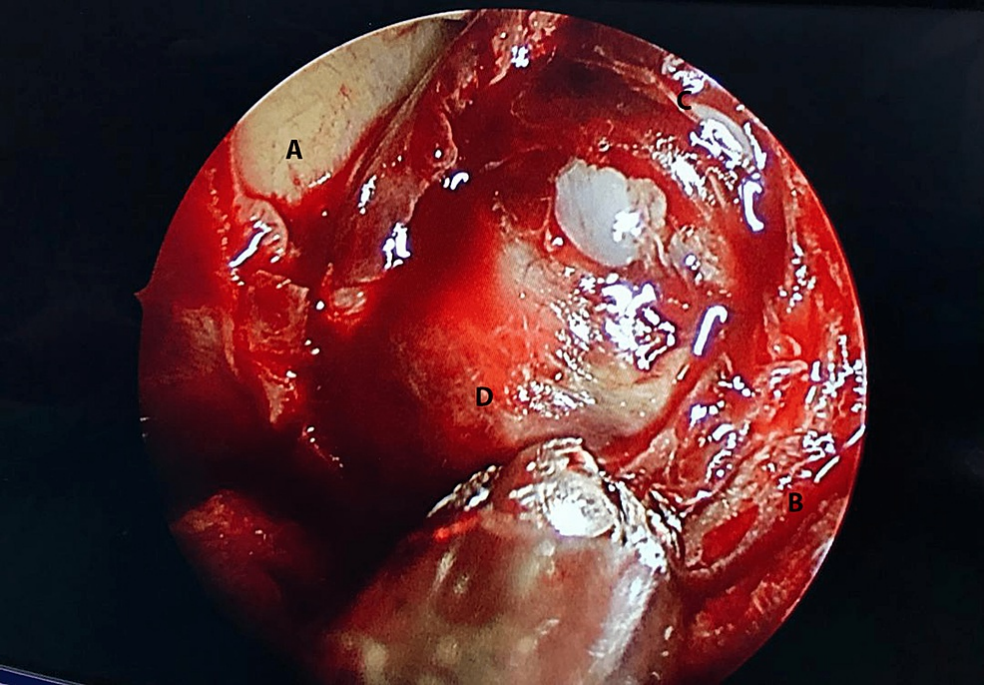
Endoscopic view of the well-encapsulated right sphenoid mass through a naturally enlarged sphenoid ostium. A) Right orbital wall, B) Nasal septum, C) Enlarged sphenoid ostium, D) Sphenoid tumor.

There was tumor invasion into the cavernous sinus and encasement of the internal carotid arteries. Extension into the pituitary fossa was noted, displacing the pituitary gland and stalk to the left. An impingement on the right optic nerve proximal to the optic chiasm, close to the orbital fissure, was also apparent. Additionally, there was meningeal thickening and enhancement in the right anterior temporal region, suggestive of tumor infiltration. A right functional endoscopic sinus surgery was performed, and a biopsy of the sphenoid mass was taken. Intraoperative findings revealed a polypoidal mass occupying the whole sphenoid sinus with extension into the right posterior ethmoid (Figure 2).

**Figure 3:**
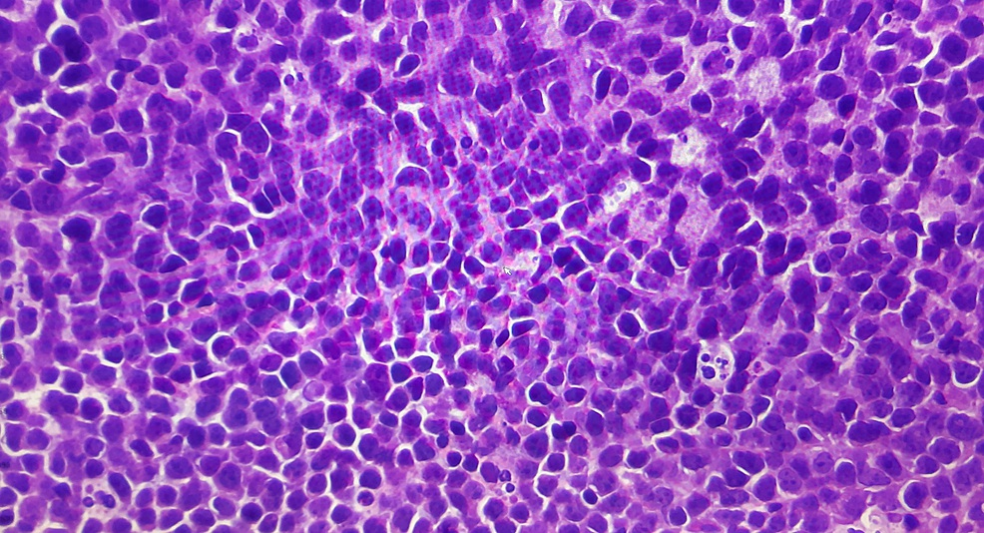
Microscopic images (100x magnification) of H&E stained tumor tissue composed of diffuse sheets of malignant lymphoid cells.

Histopathology of the mass was suggestive of a high-grade B cell lymphoma (WHO Classification 2017, 4th edition) [3]. The tumor tissue showed diffuse sheets of malignant lymphoid cells with very few admixed small lymphocytes and some prominent starry sky macrophages (Figure 3).

**Figure 4:**
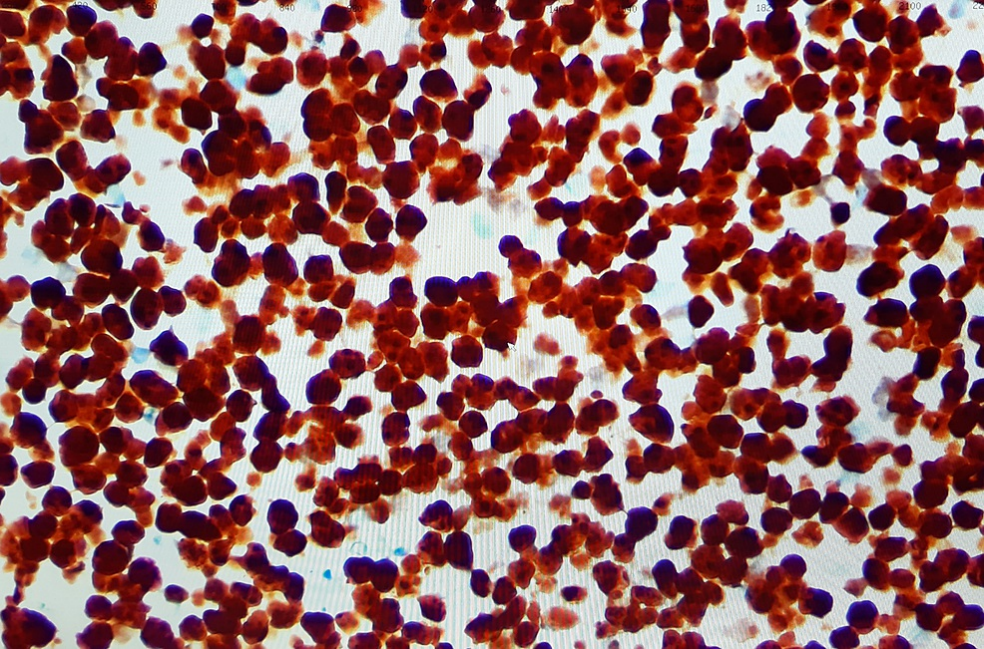
Microscopic image (100x magnification) showing tumor cells expressing immunostain for B lymphoid cells using the PAX5 nuclear stain.

The tumor cells were medium-sized, exhibiting hyperchromatic nuclei, round to irregular nuclear membrane, clumped chromatin, some with prominent nucleoli, and active mitosis with the ki67 proliferative index of 100%. The tumor cells expressed CD10, CD20, CD79a, MUM 1, BCL2, BCL 6, C MYC, and nuclear stain PAX 5 (Figure 4).

Prior to the commencement of treatment, her condition continued to worsen with the onset of cervical lymphadenopathy with orbital apex syndrome secondary to the sphenoid mass. The patient received a chemotherapy regime of R-R1 protocol and R-R2 protocol alternating for three cycles. The regime included a combination of 6-mercaptopurine, vincristine, methotrexate, cytarabine, and daunorubicin. The patient's response to treatment was excellent, with tumor regression and partial restoration of right eye vision.

## Discussion

The paranasal sinus is a rare site for primary malignancy, accounting for less than 1% of all cancers, with the sphenoid sinus being the least common among the four sinuses to contribute to these cases [4]. The malignancy types affecting the sphenoid sinus include squamous cell neoplasm, adenocarcinomas, non-Hodgkin's mature B-cell lymphoma, and unspecified epithelial neoplasm [4]. Sphenoid lymphomas are unique in their clinical presentation as compared to malignancies in the other sinuses. Headache and cranial neuropathy were the presenting symptoms in this case, followed by visual disturbances. B-cell lymphomas tend to occur in sinuses free of nasal diseases, whereas peripheral T-cell lymphoma and natural killer/T-cell lymphoma occur primarily in the nasal cavity [5]. B-cell lymphoma of the sphenoid sinus in our patient exhibits an aggressive nature, with histology showing tumor cells with a high proliferation index and active mitosis. In contrast, the MRI showed evidence of extensive involvement of surrounding structures. We noted an accelerated clinical deterioration in our patient from the time of diagnosis, likely attributed to anatomical factors and the aggressive nature of B-cell lymphomas in the paranasal sinuses. The sphenoid sinus has a vital position at the apex of the nasal cavity. It lies in close proximity to the optic canal, dura mater, pituitary gland, and cavernous sinus, which contains the internal carotid arteries and the third, fourth, fifth, and sixth cranial nerves [6]. Therefore, the progression of this malignancy will lead to compression, infiltration, or encasement of these important neighboring structures and, thus, its devastating effects, as seen in our case.

Diagnostic challenge

Due to their neurological and orbital presentations, sphenoid malignancies remain a diagnostic challenge for Otorhinolaryngologists. A high degree of suspicion and robust collaboration between teams are essential for the early detection of these lesions. Access to imaging technology also significantly influences the early detection of these lesions, many of which are found incidentally. Endoscopic sinus surgery provides a pathway toward faster diagnosis for various types of sphenoid lesions. However, it presents a challenge for rhinologists, as they must walk a fine line between obtaining adequate tissue samples to confirm a diagnosis and avoiding damage to the critical surrounding areas. Orbital apex syndrome is characterized by ophthalmoplegia, ptosis, proptosis, positive relative afferent pupillary defect, and visual impairment due to involvement of cranial III, IV, and VI secondary to sinusitis, tumor, or inflammation [7]. The degree of eyelid ptosis and vision improved drastically after removing the postoperative nasal packing. This can be explained by postoperative edema and hematoma, which further impinged the optic and oculomotor nerve. Minimally invasive biopsies have caused sudden vision loss in some instances of sphenoid lymphoma [8]. An added advantage of this endoscopic sinus surgery is that it opens a window in the sphenoid sinus, permitting tumor monitoring and response to treatment by simple nasoendoscopy.

## Conclusions

A high clinical degree of suspicion and good collaboration between primary care, ophthalmology, and otorhinolaryngology teams are pivotal in the early diagnosis of sphenoid lymphoma. Early symptoms such as facial numbness or visual disturbances should not be dismissed, and thorough clinical examination is essential. The aim is to detect sphenoid malignancies before they reach a considerable size and before they cause irreversible damage.
